# Governing AI for Mental Health: Fragmented State Approaches and the Case for a Federal Framework

**DOI:** 10.2196/96389

**Published:** 2026-07-28

**Authors:** Abir Aldhalimi

**Affiliations:** 1 Yale University School of Medicine New Haven, CT United States; 2 The Mental Health AI Policy Project Washington, DC United States

**Keywords:** artificial intelligence, AI, mental health, policy, mental health policy, AI regulation, chatbot, legislation, law, generative AI, health care policy, AI governance

## Abstract

State-level regulation of AI used for mental health is emerging in the absence of a federal framework. States are taking different approaches to regulation, resulting in a fragmented regulatory landscape. This Viewpoint aims to identify the governance approaches that US states are using to regulate the use of AI in mental health and analyze the limitations of each. A 4-state case analysis was conducted using the statutory text of bills and laws in Illinois, Utah, New York, and Nevada. Two governance approaches were identified. The first regulates the use of AI in clinical contexts, and the second regulates the technology itself. Some states have combined elements of both approaches to address AI use more comprehensively. While these approaches aim to mitigate harm, they differ in where they believe risk lies in the use of AI for mental health support. The limitations of these divergent approaches include uneven protections for consumers and regulatory uncertainty for developers, vendors, deployers, and clinicians. Because AI in mental health operates across both clinical and consumer domains, neither approach alone can address the risks associated with its use for mental health support. A coordinated, risk-based federal regulatory floor is needed to ensure consistent protections across states.

## Introduction

Mental health conditions are on the rise in the United States, particularly among young people [1]. The current system is not capable of meeting the country’s growing mental health needs, especially in light of a behavioral health care provider shortage [2]. Consequently, approximately 40% of adolescents who need mental health care receive no treatment [3]. AI may help address these gaps for clinicians, patients, and consumers.

The release of OpenAI’s GPT-3.5 in 2022 led to widespread and rapid adoption of AI for mental health support. A US survey of 1058 adolescents and young adults found that 13% had used generative AI for mental health advice; of those, 65% did so monthly, and 93% found the advice helpful [4]. The adoption may reflect the technology’s accessibility and affordability, as well as its ability to produce humanlike conversational responses that create an impression of connection. At the same time, AI presents significant risks. These include data breaches, reinforcement of harmful beliefs, and hallucinated outputs [5,6]. In some instances, the use of AI for mental health support has been associated with serious harm, including suicide [7]. These cases have raised concerns about whether these tools have adequate safeguards.

In response to these concerns, US states have begun to introduce laws governing the use of AI in mental health. In the absence of a unified federal regulatory policy, states have taken divergent approaches to regulating this domain. This paper identifies and analyzes these approaches, clarifying the current policy landscape and highlighting where they differ. These approaches, while descriptively useful, cannot serve as a normative framework for future regulation because AI for mental health support operates across multiple domains, including clinical and consumer use. A single regulatory approach will therefore overlook certain risks associated with the use of AI in mental health, and variation across states will lead to uneven protections. For these reasons, this paper calls for a federal, risk-based regulatory framework.

To examine how states are regulating AI in mental health, this analysis draws on the statutory text of bills and laws. Each state’s official legislative website was searched for AI-related mental health legislation. New York, Illinois, Utah, and Nevada were selected for case study analysis because they were among the first states to introduce or enact such policies. Each bill or law was reviewed in full to confirm that its focus was on AI and mental health. Broader state AI laws that apply to mental health were not the primary focus of this analysis.

## Governance Approaches

Two main approaches emerged in this analysis. The first regulates AI’s use in clinical contexts, referred to here as the “clinical governance approach.” The second regulates the technology itself, referred to here as the “consumer protection approach.” Some states have combined elements of both approaches to address AI use more comprehensively. Each approach recognizes that AI in mental health carries risks. They differ, however, in where they place risk and who is held accountable when harm occurs. Each approach, including the hybrid one, has limitations, which the case studies below examine.

The clinical governance approach reaffirms that psychotherapy is a protected professional activity and that AI introduces clinical risk when used by licensed professionals. Regulators place responsibility on clinicians when AI-related harm occurs during treatment. The laws restrict clinicians’ use of AI in diagnosis and treatment planning, clinical recommendations, and therapeutic communication. While the use of AI in clinical decision-making is prohibited, a clinician may use AI for administrative or supplementary tasks; however, these tasks are narrowly defined. The approach also imposes strict informed consent requirements. Clinicians must obtain separate written consent for AI use, and patients may withdraw consent at any time. Agencies that regulate clinical professional practice enforce these laws. Illinois has adopted this approach to regulating AI in mental health.

The consumer protection approach treats AI-based mental health tools such as chatbots and AI companions as consumer products, placing responsibility for risk on developers, vendors, and deployers. This approach does not necessarily regulate the clinical or therapeutic content of the tool itself. Rather, regulators focus on the potential harm the tool might cause through deception, fraud, or misrepresentation. Developers, vendors, and deployers may be required to mitigate risk through safety protocols, user disclosures, and restrictions on deceptive marketing. However, there is limited consensus on which safeguards to require in statutes, resulting in uneven requirements from one state to the next. In this approach, attorneys general and consumer protection agencies have enforcement responsibility. Utah has adopted this approach.

Each approach addresses distinct issues that may arise from the use of AI in mental health. As a result, both approaches leave some aspect of the problem unregulated. For example, the consumer protection approach does not assess clinical safety, and the clinical governance approach does not regulate the tools that consumers would use outside clinical settings. AI use in mental health is complex to regulate because it spans multiple domains: clinical interventions, wellness apps, chatbots, and AI companions. It also raises concerns about clinical safety, data security, and consumer protection. Some states have adopted a hybrid approach combining elements of both to better address these risks. New York and Nevada are 2 examples. While both states incorporate clinical and consumer protection elements, their laws are structured differently, as discussed below.

## State Law Case Studies

### Clinical Governance Approach

In August 2025, Illinois enacted the Wellness and Oversight for Psychological Resources Act (HB1806) [8]. The law is a clear example of the clinical governance approach. While the law aims to protect patients by regulating the clinicians’ use of AI in clinical practice, it has notable limitations. For example, the law explicitly excludes physicians from its definition of a licensed professional even though it applies to a wide range of other mental health care providers. This omission creates uneven liability for clinicians delivering overlapping mental health services. In practice, a psychologist and a psychiatrist may see the same patient, yet only the psychologist will face greater liability if they use an AI tool to assist with treatment. In clinic or hospital settings, this law will require health institutions to decide how to address these uneven requirements to remain compliant with the law.

Clinicians are rapidly adopting AI in clinical practice. In a recent American Psychological Association survey of 1742 psychologists, 56% reported using AI to assist in their practice at least once in the previous year [9]. Mental health care providers in Illinois who are unfamiliar with the law’s restrictions face greater liability, including disciplinary action by the licensing board and potential malpractice exposure if a clinician’s misuse of AI contributes to patient harm. Enforcement is carried out by the Department of Financial and Professional Regulation, which may impose a civil penalty of up to US $10,000 per violation.

This statute also places a greater burden on licensed practitioners without providing them with the support needed to ensure compliance. Clinicians must determine for themselves whether their use of AI violates the law. This may be especially difficult because clinicians are rarely trained on these tools, nor are they involved in their design and deployment. Furthermore, licensing boards, often made up of practicing clinicians and public members, may lack the technical expertise to assess whether the use of AI violates the law. This will raise the prospect of inconsistent enforcement and contribute to clinicians’ uncertainty about AI use. As a result, mental health clinicians may become reluctant to use AI support in treatment even when it is allowed. Given the ongoing behavioral health care workforce crisis, this governance approach could hinder efforts to address workforce shortages and clinician burnout.

Finally, Illinois’s law defines AI use narrowly, with no exceptions for Food and Drug Administration (FDA)–approved tools even though the latter regulates these tools as medical devices. Essentially, under this law, a vetted medical device for mental health is considered as risky as an unvetted one. These shortcomings will have consequences for both mental health clinicians and patients.

### Consumer Protection Approach

Utah has adopted a consumer protection approach, framing AI-related mental health risks as matters of consumer safety and holding developers, deployers, and vendors accountable. Utah’s law regulating mental health chatbots (HB452) [10] took effect in May 2025. While Illinois regulates the clinician’s use of AI, Utah regulates the technology itself without banning it. It requires mental health chatbots to provide clear disclosure, bans the sale of identifiable health data, and restricts advertising. The law also creates an affirmative defense framework. It provides liability protections for developers, vendors, and deployers who adopt these clinical safety protocols. This essentially makes the law’s clinical safety measures voluntary. The Utah Division of Consumer Protection enforces this law, with civil penalties of up to US $2500 per violation.

Utah’s law does not address the content of these tools. By doing so, the law leaves key design decisions related to mental health to AI developers. For example, while the law’s affirmative defense framework asks developers, deployers, and vendors to describe how licensed mental health care providers are involved in the technology’s development, it does not define what constitutes meaningful clinical involvement. As a result, developers can satisfy this requirement with minimal input from mental health professionals. Furthermore, those who do not pursue the affirmative defense are not required to describe the clinical involvement at all. Such leeway leaves clinical adequacy in the hands of these parties, largely unregulated. This matters because the consumers of these chatbots include vulnerable groups, such as children and youth, individuals with serious mental illness, and those without access to professional mental health services. For users in crisis or with serious mental health needs, poorly designed tools can cause real harm.

### Hybrid Approach

New York and Nevada both recognize that AI in mental health requires regulating both clinician use and consumer-facing AI technologies. However, the 2 states structure their laws differently. Nevada has adopted a hybrid approach by integrating clinical and consumer protection into a single statute. New York addresses both approaches through 2 statutes: a consumer protection statute and a clinical governance bill that is still pending. New York uses 2 enforcement bodies, including the attorney general, a general-purpose enforcement office. Nevada’s enforcement is carried out by agencies with mental health expertise, including the Division of Public and Behavioral Health and the relevant licensing boards for clinicians. Differences among enforcement bodies may affect how each state evaluates the clinical risks of AI products.

New York’s Artificial Intelligence Companion Models Law [11], which took effect in November 2025, applies a consumer protection framework to AI companions. AI companions are technologies that provide ongoing emotional and relational support through humanlike interaction. They are often marketed for general rather than clinical use. New York’s law, similar to Utah’s, requires disclosure that users are interacting with an AI tool. It further mandates that the disclosures appear at the start of each interaction (no more than once per day) and recur every 3 hours during extended use. Unlike Utah, New York requires operators of AI companions to implement safety protocols, including referring users to appropriate care when detecting suicidal thoughts or risk. Similar to Utah, enforcement falls within consumer protection. The attorney general oversees this law, with penalties of up to US $15,000 per day for a violation.

Although not yet enacted, New York’s Oversight of Technology in Mental Health Care Act [12] aligns with the clinical governance approach by adopting a model similar to Illinois’s. As in Illinois, psychotherapy is a protected activity to be delivered by a licensed mental health professional. The commissioner of education, who oversees professional licensing for mental health care providers in New York, would enforce this law. Violations would be subject to civil penalties of up to US $50,000 per violation. If enacted, the bill would mirror the shortcomings of Illinois’s law, including the exclusion of physicians. In addition to these drawbacks, the structure of New York’s 2 statutes may create fragmentation in enforcement, increasing the risk that some AI use in mental health will fall between the 2 bodies and go unregulated.

Nevada’s AB406 law [13], which took effect in July 2025, regulates AI in mental health across AI providers, clinicians, and public schools. The Division of Public and Behavioral Health within Nevada’s Department of Human Services enforces violations by AI providers. The relevant licensing board addresses clinician misuse as unprofessional conduct. Civil penalties for product violations can reach up to US $15,000 per violation.

Unlike Illinois’s law, Nevada’s explicitly includes psychiatrists, ensuring that liability applies consistently across mental health clinicians who deliver overlapping services. Furthermore, Nevada’s law extends beyond the clinical setting to include clinicians working in schools. The law prohibits AI from performing the functions of a school counselor, psychologist, or social worker. It also directly regulates AI providers, reflecting its hybrid approach. Specifically, it prohibits AI providers from offering AI tools that would deliver mental health care. These provisions aim to prevent deceptive practices that could lead consumers to believe they are receiving clinical care.

## Consequences of a Fragmented Governance Landscape

The emergence of these governance approaches has produced a fragmented regulatory landscape, with significant consequences for consumers, patients, clinicians, developers, vendors, and deployers. Analyzing the statutes across these 4 states highlighted several challenges resulting from this fragmentation.

### Uneven Protection for All Stakeholders

The current patchwork of state laws results in uneven protection for individuals using AI for mental health support. In Utah, users of mental health chatbots receive specific consumer protections. In contrast, individuals in states without consumer protection laws may have little or no protection when using similar tools. This gap is especially clear in a state that only regulates AI through a clinical governance approach. In those states, clinicians’ use of AI is regulated, but patients can leave the clinical setting and use unregulated chatbots for mental health support.

The variability also affects developers, deployers, and vendors. Those operating across state lines must adapt their products to comply with different and sometimes conflicting regulations. For example, a chatbot designed to provide therapeutic mental health support in Utah would require adjustments to operate legally in Nevada, where AI cannot provide professional mental health care.

### Regulatory Burden on Clinicians Across State Lines

AI is increasingly integrated into electronic health record (EHR) systems used across health care settings. In integrated care settings, mental health clinicians have limited control over the technologies they are required to use in clinics or hospitals. Furthermore, they may not even know which AI tools are embedded in EHRs. These laws would then hold clinicians accountable for the systems often chosen for them. In practice, a tool permitted to a physician may pose legal risk to a mental health care provider using the same system, as in Illinois’s case. Furthermore, EHR systems are deployed across state lines, which means that a clinician seeing patients in multiple states faces additional legal risk.

Mental health clinicians are increasingly practicing across state lines, driven by the expansion of the Psychology Interjurisdictional Compact (PSYPACT). As of 2026, a total of 46 jurisdictions have enacted PSYPACT legislation, and it is effective in 43 [14]. PSYPACT allows licensed psychologists to practice across participating states through telepsychology and limited in-person services. Illinois and Nevada are among them. New York is poised to join. Differences in state AI regulations mean that mental health clinicians practicing across states through PSYPACT must navigate conflicting requirements, adding a new layer of burden to cross-state practice.

## Lessons From Other Mental Health Policy Areas

This policy fragmentation has real consequences. While AI’s proliferation in mental health is new, there are lessons to be learned from other mental health policy areas. Mental health parity has seen similar state-level fragmentation. At the federal level, the Mental Health Parity and Addiction Equity Act required insurers offering mental health and substance use coverage to do so on par with medical and surgical services [15]. While parity was mandated under federal law, enforcement authority was divided between federal agencies and state regulators. This resulted in states diverging substantially in how they enacted and enforced parity, producing uneven access to care for patients and inconsistent reimbursement standards for the behavioral health workforce [16].

The same dynamic will likely play out in the AI and mental health space. Without a federal framework, or with one that gives states too much leeway in defining and enforcing laws in this domain, fragmentation will continue. This will result in significant drawbacks for consumers and patients; additional liability for an already strained mental health workforce; and limitations on innovation for developers, deployers, and vendors. A coordinated federal regulatory framework is necessary.

## Federal Regulatory Uncertainty

Federal policy adds another layer of complexity. In December 2025, an executive order titled “Ensuring a National Policy Framework for Artificial Intelligence” [17] directed the creation of an AI Litigation Task Force within the Department of Justice. The task force is charged with investigating and, if necessary, challenging state laws that impede federal AI priorities. The executive order may challenge state laws regulating the use of AI in mental health if the task force deems them excessive regulation of AI. This is already playing out. The order set the stage for Colorado’s first AI law to be challenged in court [18]. In response, Colorado is replacing its first AI law with the scaled-back SB189 [19]. The same could happen in Utah, New York, and Nevada, particularly if their safety protocol requirements are deemed excessive. As a result of this federal action, states may become reluctant to regulate AI use in mental health.

The executive order demonstrates the current administration’s position on AI regulation, which has gone so far as to challenge state-level AI laws. It signals the federal interest in shaping AI policy but does so by limiting state authority rather than establishing national standards. The executive order fails to set clear requirements for AI safety, ethical use, or national governance. Regulatory uncertainty is likely to continue without a comprehensive federal framework.

## The Need for a Federal Regulatory Framework

A recent 50-state legislative review of AI in mental health identified 4 themes across the bills and laws: professional oversight, harm prevention, patient autonomy, and data governance [20]. These themes span the governance approaches discussed in this paper. Regulating AI in mental health is multifaceted, and approaches limited to a single regulatory framework are likely ineffective.

A regulatory framework can help close the gaps noted in the bills and laws discussed in this paper. However, a federal framework cannot extend to every domain. The clinical scope of practice has historically been regulated by states through licensing boards, and a federal action cannot displace that authority. Instead, a framework would regulate consumer protection, medical device oversight, and health data standards. These are areas that have historically been regulated at the federal level. Building on this authority, a framework could establish a federal floor for AI tools and coordinate across jurisdictions, and states can continue to build additional protections. The framework would help reduce inconsistencies and confusion by standardizing baseline protections across states.

Within these federally regulated domains, the federal framework would need to distribute accountability across the technology’s design, development, and deployment. The United States could achieve this by adopting a risk-based federal regulatory framework that incorporates evidence-based safeguards. The US government has already produced guidance that can be used as a starting theoretical framework. Under the National Institute of Standards and Technology’s (NIST) AI risk management framework [21], AI risk is defined by both the likelihood and severity of harm within a given context. In mental health, that risk depends on how the technology is designed, developed, and deployed; how users engage with it; and the user’s condition. The NIST’s framework offers a useful starting structure for federal policy in this area; however, its adoption is voluntary. It is critical that a federal framework make these standards mandatory either by statute or by rule rather than leaving them voluntary, so that they can be enforced.

Accordingly, not all AI applications in mental health pose the same level of risk, so a one-size-fits-all regulatory approach is likely ineffective. Risk should be based on the tool’s function and context. Aligned with the NIST’s approach, the European Union’s (EU) AI Act classifies technologies based on their potential harm and requires different levels of oversight depending on risk [22]. The EU AI Act establishes 4 risk categories: prohibited, high risk, limited risk, and minimal risk. This risk-based approach is particularly applicable to AI tools used in mental health as the tools can vary widely in function and potential harm.

Under the EU AI Act risk-based tiers, a wellness app used for mindfulness or stress management may carry minimal risk. High-risk tools in mental health may include chatbots offering therapeutic advice and tools designed to deliver clinical interventions. Similarly, tools that listen to and transcribe therapeutic conversations may be categorized as high risk due to data and privacy concerns. AI companions that influence users’ emotions may fall under the prohibited category if they use manipulative or deceptive techniques. This risk-based categorization is useful because higher-risk AI technologies receive stronger protections.

Translating this risk-based approach into practice requires a clear enforcement structure. Because mental health touches on several domains, a federal framework would require multiple agencies to work together. The US Department of Health and Human Services (HHS) would lead on health-related issues, including enforcement of health data and HIPAA (Health Insurance Portability and Accountability Act) provisions. Within the HHS, the Substance Abuse and Mental Health Services Administration would provide expertise on mental health policy. The Federal Trade Commission would regulate consumer protection. The FDA already regulates AI mental health tools that function as medical devices and can continue to do so under a federal framework. FDA approval can inform which tools states allow clinicians to use in practice while the decision itself remains with state licensing authorities.

Coordinating across these agencies is difficult without a central body, and regulating AI for use in mental health will likely require one. The Office of the National Coordinator for Health IT within the HHS offers a potential model. It is a congressionally mandated office that coordinates government-wide efforts on health IT policy. A similar coordinating office focused on AI in mental health could ensure consistent implementation of federal policy, support state efforts to build additional protections, and help coordinate across federal and state regulators.

## Limitations

This analysis is limited to 4 states that have enacted or introduced legislation regulating the use of AI in mental health support. This is a fast-moving regulatory landscape, and states may introduce additional legislation that may add to or challenge the governance approaches documented in this paper. Another limitation of this paper is its focus on bills and laws that specifically regulate AI and mental health as it does not examine the broader AI statutes that apply to mental health.

## Conclusions

AI became widely available to the public only a few years ago, and its impact on society continues to unfold. Its adoption in mental health is among the most striking and least anticipated developments. There is a strong likelihood that the public will continue to use AI for mental health support, and the risks are becoming better documented. Evidence-based regulation is necessary. States have begun to regulate this nascent policy area, although their approaches are still taking shape. The current analysis reveals that states are taking divergent approaches to regulating this domain, with some regulating the technology itself and others regulating its use in clinical practice. States are regulating a variety of AI tools, including companion and therapy chatbots. Each of these tools carries distinct risks, and a single regulatory approach may not address them all. While some states are adopting hybrid approaches, the resulting regulations remain inconsistent. Without a national risk-based, evidence-informed framework, well-intentioned state efforts to protect consumers and patients may continue to produce uneven protections. How policymakers both at the federal and state level address this challenge will shape the safe and effective use of AI in mental health at a time when the United States is experiencing a significant mental health crisis.

## Abbreviations

EHR: electronic health record
EU: European Union
FDA: Food and Drug Administration
HHS: US Department of Health and Human Services
HIPAA: Health Insurance Portability and Accountability Act
NIST: National Institute of Standards and Technology
PSYPACT: Psychology Interjurisdictional Compact

## References

[1] Mental illness. National Institute of Mental Health.

[2] (2023). The behavioral health care workforce. NIHCM Foundation.

[3] (2024). Key substance use and mental health indicators in the United States: results from the 2023 National Survey on Drug Use and Health. Substance Abuse and Mental Health Services Administration.

[4] McBain RK, Bozick R, Diliberti M, Zhang LA, Zhang F, Burnett A, Kofner A, Rader B, Breslau J, Stein BD, Mehrotra A, Pines LU, Cantor J, Yu H (2025). Use of generative AI for mental health advice among US adolescents and young adults. JAMA Netw Open.

[5] Nutbeam D, Milat AJ (2025). Artificial intelligence and public health: prospects, hype and challenges. Public Health Res Pract.

[6] Frances A (2026). Warning: AI chatbots will soon dominate psychotherapy. Br J Psychiatry.

[7] Head KR (2026). Digital companions, real casualties: a commentary on rising AI-related mental health crises. Curr Res Psychiatry.

[8] (2025). Wellness and Oversight for Psychological Resources Act, Public Act 104-0054. State of Illinois.

[9] (2025). AI in the therapist’s office 2025: Practitioner Pulse Survey. American Psychological Association.

[10] (2025). Artificial Intelligence Amendments, HB 452. 2025 General Session, State of Utah.

[11] (2025). Artificial Intelligence Companion Models Law, S3008-C, 2025-2026 Legislative Session, New York. New York State Senate.

[12] (2025). Oversight of Technology in Mental Health Care Act, S8484, 2025-2026 Legislative Session, New York. New York State Senate.

[13] (2025). Assembly Bill 406, 83rd Legislative Session, Nevada. State of Nevada.

[14] PSYPACT map. Psychology Interjurisdictional Compact Commission.

[15] Barry CL, Huskamp HA, Goldman HH (2010). A political history of federal mental health and addiction insurance parity. Milbank Q.

[16] (2024). Mental health and substance use disorder insurance parity: summary of state laws. Legislative Analysis and Public Policy Association.

[17] (2025). Ensuring a national policy framework for artificial intelligence. The White House.

[18] (2026). Justice department intervenes in xAI lawsuit challenging Colorado’s ‘Algorithmic Discrimination’ Law. Office of Public Affairs U.S. Department of Justice.

[19] SB26-189: automated decision-making technology. Colorado General Assembly.

[20] Shumate JN, Rozenblit E, Flathers M, Larrauri CA, Hau C, Xia W, Torous EN, Torous J (2025). Governing AI in mental health: 50-state legislative review. JMIR Ment Health.

[21] (2023). Artificial Intelligence Risk Management Framework (AI RMF 1.0). National Institute of Standards and Technology.

[22] The EU Artificial Intelligence Act. Future of Life Institute.

